# Wafer‐Scale Near‐Infrared Metalenses With High Performance and Low Cost

**DOI:** 10.1002/nap2.70258

**Published:** 2026-08-18

**Authors:** Wanjiao Zhang, Meng Wang, Yunxiao Cai, Qiu Wang, Yuan Wang, Lu Han

**Affiliations:** ^1^ Hangzhou Institute for Advanced Study University of Chinese Academy of Sciences Hangzhou China; ^2^ School of Information Engineering and the Hubei Key Laboratory of Broadband Wireless Communication and Sensor Networks Wuhan University of Technology Wuhan China; ^3^ State Key Laboratory of Optical Quantum Materials Department of Mechanical Engineering The University of Hong Kong Hong Kong China

**Keywords:** all‐dielectric, metalenses, nanoimprinting lithography, wafer‐level

## Abstract

Refractive optics, now over two centuries old and highly mature, remains the cornerstone of optical systems in rapidly growing fields such as robotics and smart sensing, yet has reached its fundamental physical limits. Ultra‐thin metalenses hold transformative potential for next‐generation optics, yet their widespread adoption is severely impeded by the high cost of wafer‐level fabrication. In this paper, 8‐inch wafer‐scale near‐infrared all‐dielectric metalenses are demonstrated by a scalable high‐precision manufacturing scheme that dramatically reduces cost while ensuring exceptional uniformity and performance. Our step‐and‐repeat (S&R) imprinting strategy eliminates the need for costly deep‐UV lithography or large‐area direct‐write tools, reducing master mold costs by over an order of magnitude. Optical characterization confirms diffraction‐limited focusing performance and remarkable consistency across the entire wafer, validating both process robustness and design fidelity. Owing to its compatibility with diverse material systems and standard semiconductor processes, this methodology paves a practical high‐yield pathway toward the commercial deployment of high‐performance metasurfaces for demanding applications, including LiDAR, AR/VR, and aerospace‐grade optics.

## Graphical abstract

Widespread adoption of metalenses is constrained by prohibitive cost of wafer‐level fabrication. This work achieves 8‐inch wafer‐level fabrication with high uniformity (feature deviation < 5%) and reliable performance by step‐and‐repeat nanoimprint lithography. Owing to low‐cost advantage and compatibility with diverse material systems and standard semiconductor processes, the proposed methodology paves a practical, high‐yield pathway toward the commercial deployment of high‐performance metasurfaces.

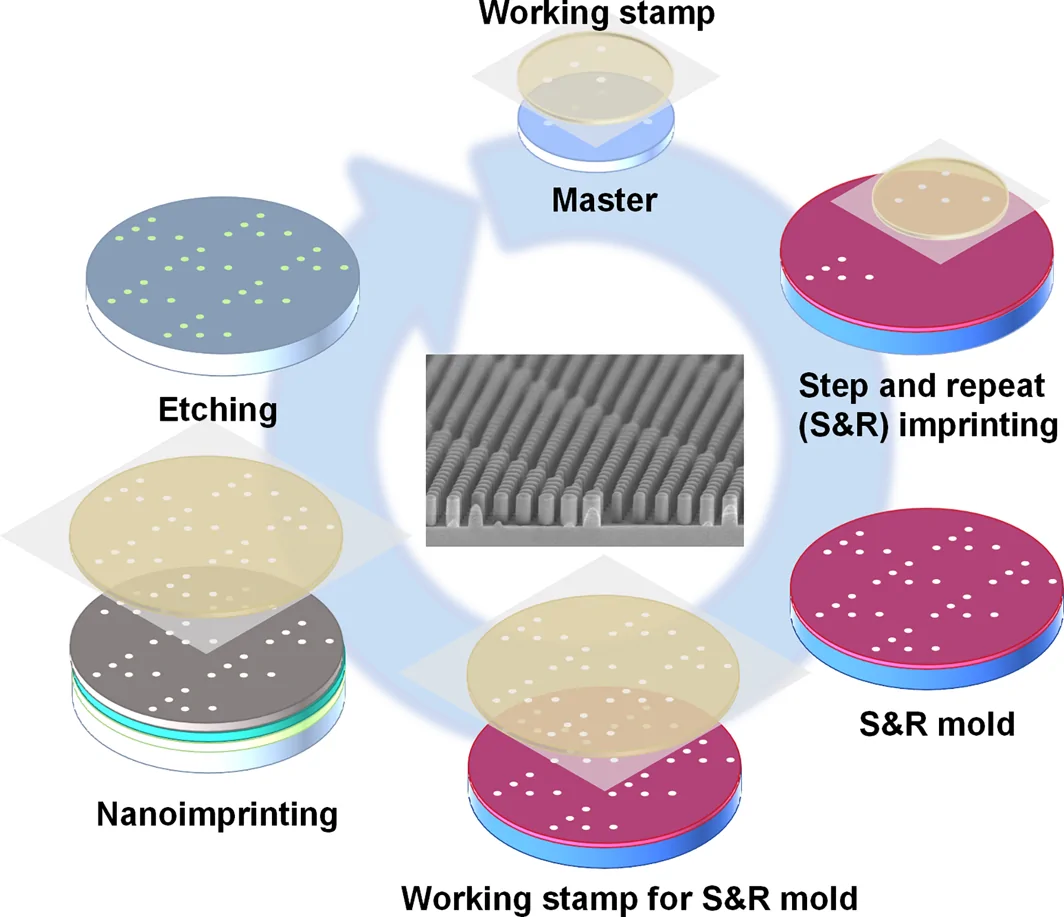

## 1 Introduction

Boasting a 200‐year development history and currently standing at the peak of technical maturity, refractive optical technology remains the dominant core technology underpinning optical components in fast‐emerging sectors. However, after centuries of iterative optimization, this conventional optical framework has hit its inherent physical boundaries: further improvements in resolution, light efficiency, and device compactness are no longer feasible within its existing theoretical and structural paradigm, underscoring the urgent need for disruptive alternatives to propel optical technology forward.

Metalenses, ultra‐thin planar optical elements composed of subwavelength nanostructures (meta‐atoms), have garnered significant interest in both academia and industry in recent years [1, 2, 3]. By locally engineering the phase [4, 5], amplitude [6, 7], and polarization [8, 9] of light at the subwavelength scale, sometimes even simultaneously [10, 11, 12], metasurfaces transcend the limitations of conventional refractive optics, which rely on gradual phase accumulation through curved surfaces. This unprecedented light control enables multifunctional integration within a single flat device, including achromatic focusing [13, 14, 15, 16], correction of spherical and comatic aberrations [17, 18, 19], polarization multiplexing [20, 21], active tunability [22], and even nonlinear optical responses [23]. Consequently, metalenses have been actively explored for applications such as 3D sensing [24, 25], light detection and ranging (LiDAR) [26, 27, 28], spectral sensing/imaging [29, 30], and augmented/virtual reality (AR/VR) near‐eye displays [31, 32, 33], with several prototypes already demonstrating tangible improvements in system performance and miniaturization.

Despite this rapid progress, wafer‐scale metalenses with high stability and uniformity create substantial fabrication challenges, which currently are also the primary bottleneck to their widespread industrial adoption. To date, two lithographic approaches have been pursued for large‐scale fabrication: deep ultraviolet (DUV) lithography [34, 35, 36] and Nanoimprint Lithography (NIL) [37, 38, 39]. Metalenses have been successfully fabricated on 4‐inch fused silica wafers via DUV lithography combined with dry etching, but the high cost of DUV equipment and its incompatibility with transparent substrates hinder their broader applications. In this context, NIL has attracted increasing research interest as a scalable manufacturing approach for metalenses. Numerous studies have demonstrated various functionalities of NIL‐fabricated metalenses, including holographic imaging and multifocal focusing, among others [40, 41]. Furthermore, NIL‐fabrication of metalenses spanning from 8‐inch wafer‐scale to large‐format flexible substrates (300 mm × 200 m) has been successfully demonstrated, highlighting its potential for low‐cost and high‐throughput production of flat optical devices [42, 43, 44]. Most of these demonstrations are based on single‐step NIL, where polymer imprint resins directly define the optical nanostructures, offering a simplified fabrication route. However, such polymer‐based implementations inherently limit the optical performance and environmental stability of metalenses. The relatively low refractive index of polymer‐based resins (typically < 2.0) restricts the achievable phase modulation range and design flexibility, while their organic nature leads to inferior thermal stability and environmental durability compared with inorganic optical materials. These limitations become increasingly critical for high‐performance metalenses targeting demanding applications such as automotive, aerospace, and harsh‐environment optical systems.

Moreover, a key challenge for NIL‐based metalens manufacturing lies in the fabrication of low‐cost, wafer‐level master molds. Since NIL molds require 1:1 replication of the target metalens, the master must faithfully define features with critical dimensions (CDs) typically below 180 nm. Fabricating such masters over large areas via electron beam lithography (EBL) is prohibitively slow and expensive [45], whereas DUV lithography requires substantial investment in exposure tools, photomasks, and process development.

To overcome these intertwined challenges, this paper presents a series of wafer‐level near‐infrared (NIR) all‐dielectric metalenses. Specifically, metalenses consisting of amorphous silicon (a‐Si) meta‐atoms were fabricated on 8‐inch quartz wafers via the integration of NIL with conventional semiconductor processing. A step‐and‐repeat (S&R) imprinting scheme was employed to generate wafer‐scale NIL molds at a significantly lower cost compared with DUV lithography or direct‐write approaches. A 50 × 50 mm master mold prepared by EBL was replicated into an 8‐inch S&R mold with ∼10 nm pattern fidelity, ∼2 nm feature size standard deviation at the wafer scale, and excellent flatness. Combined with optimized spin‐coating conditions, this allowed uniform residual layer thickness (variation ∼10 nm) across the wafer, providing a reliable basis for subsequent etching and guaranteeing high fidelity and uniformity of the final metalenses. Optical characterization confirms that near‐infrared metalenses at different device locations all exhibit focal lengths in excellent agreement with design specifications. Moreover, these metalenses demonstrate the exceptional consistency, with full width at half maximum (FWHM) variation constrained to ∼0.15 μm across the wafer. This methodology not only significantly reduces the manufacturing cost of metalenses but also is material‐agnostic, enabling scalable production of high‐performance environmentally robust flat‐optics devices for next‐generation optical technologies.

## 2 Design Principle

Here, subwavelength a‐Si cylinders patterned on a quartz substrate were employed as basic unit cells, as shown in Figure 1a. All the cylinders were 475 nm in height and 410 nm period, and the radius ranged from 60 to 140 nm. Modeling of the electromagnetic properties of the meta‐atoms was performed using CST Microwave Studio based on the above dimensions. Periodic boundary conditions were applied in both the *x* and *y* directions, whereas the perfectly matched‐layer (PML) boundary condition was applied in the *z* direction. Normally, incident *x*‐polarized plane waves with 940 nm wavelength were used to excite the structures, and a probe was set behind the structure to detect the *x*‐polarized component of the transmitted electric field. As shown in Figure 1b, within the above parameters, the phase shift range is close to 2π, whereas the transmission remains high (nearly 0.95) and stable. Nearly identical results were obtained for y‐polarized incidence (see Supporting Information S1: Figure S1).

**FIGURE 1:**
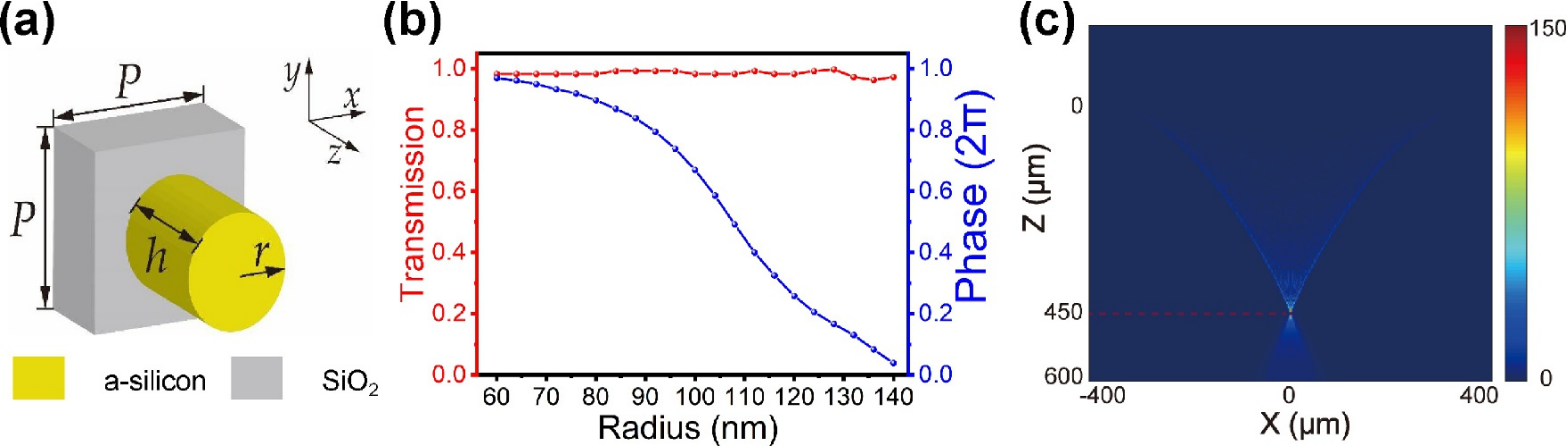
Illustration and simulated results of the metalens. (a) Schematic of an a‐Si cylinder meta‐atom patterned on a quartz substrate. *P*, *r*, and *h* represent the period, radius, and height of the meta‐atom, respectively. (b) The simulated results of transmission and phase shift of meta‐atoms with different radii under normal plane wave incidence at 940 nm. (c) Simulated normalized intensity distribution in the *x–z* plane under normal plane wave incidence at 940 nm.

Then, the single metalens with 800 μm diameter could be constructed by the selected meta‐atoms with the following phase distribution equation:

(1) $$\varphi \left(x,y\right)=-\frac{2\pi }{\lambda_{d}}\left(\sqrt{x^{2}+y^{2}+f^{2}}-f\right),$$

where $\lambda_{d}$, *x*, *y*, and *f* represent the wavelength, spatial coordinate, and the focal length, respectively. The designed focal length is 450 μm. Numerical simulations of the transmitted electric field distributions in the *x–z* plane can be seen in Figure 1c. The simulated focal point just lies at *z* = 450 μm, showing good coincidence with the design.

## 3 Results and Discussion

### 3.1 8‐Inch Wafer‐Level Fabrication of Metalenses

Regarding the designed metalens, a master mold was fabricated on a 50 mm × 50 mm silicon substrate using EBL and inductively coupled plasma (ICP) etching, with the fabrication processes shown in Supporting Information S1: Figure S2 (Supporting Information S1, for details of the process, see Experimental Section). On the master mold, five metalens‐like patterns (nano‐pillars with the same diameters as the designed metalens and a depth of ∼250 nm) were distributed within a 50 mm × 50 mm square region at the center. The 250 nm etching depth was selected based on two considerations: a relatively low aspect ratio (max. ∼2:1) for easy demolding during the NIL process and a sufficient depth to serve as a mask for etching the underlying hard mask layer (150 nm‐thick SiO_2_).

Using this master mold, an 8‐inch S&R mold was fabricated through seven step‐and‐repeat imprinting cycles, as shown in Figure 2a (for details of the process, see Experimental Section). Owing to the high‐precision positioning stage and collimated UV source of the S&R imprinting system (GL300 SR‐F, GermanLitho), the exposure area was confined to the contact region of the working stamp, while a minimum gap of 10 μm was maintained between adjacent imprint fields. Furthermore, the anaerobic S&R resist (GL‐HS, GermanLitho) polymerizes only when simultaneously contacted by the working stamp and exposed to UV light. These features effectively prevent unintended curing or mechanical interference in neighboring regions during the S&R process. The effectiveness of this strategy is further evidenced by the excellent agreement in both structural dimensions and optical performance across all seven imprinting steps, as discussed in Section 3.3. The optical images of the master and 8‐inch S&R mold are both shown in Figure 2b. As a result, 35 metalenses were replicated onto the 8‐inch wafer, which is 7 times compared with the original master mold. Benefiting from the combined protection of the ALD‐grown Al_2_O_3_ layer and the fluorinated anti‐adhesion monolayer (see Experimental Section for fabrication details), the S&R mold exhibited negligible structural degradation after 50 wafer‐level NIL cycles (Supporting Information S1: Figure S3). Furthermore, the surface hydrophobicity could be effectively restored through re‐deposition of the antiadhesion layer, enabling continued reuse of the mold while maintaining reliable demolding performance. These characteristics significantly reduce fabrication costs and support the scalable manufacturing of wafer‐scale metalenses.

**FIGURE 2:**
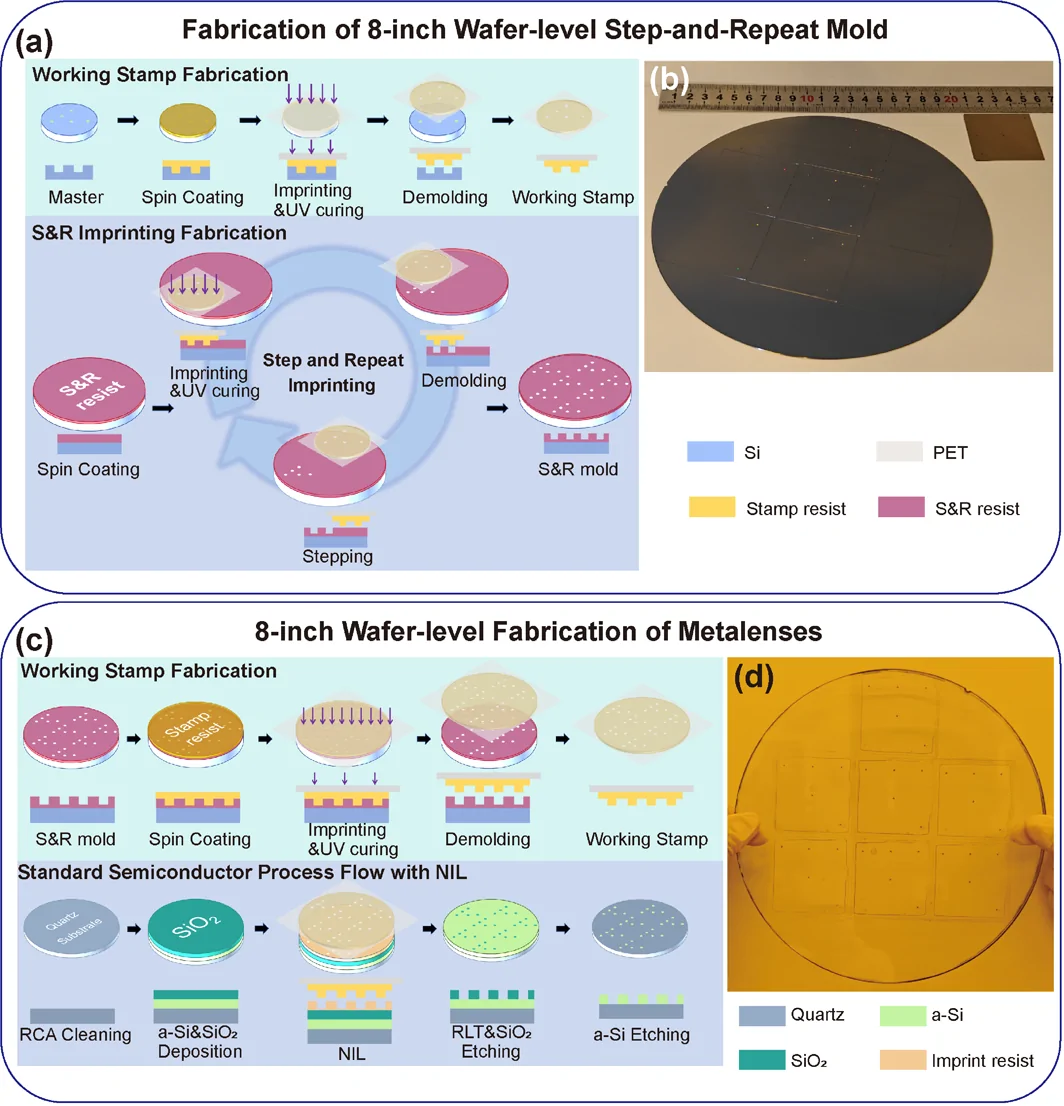
Schematic illustration of 8‐inch wafer‐level fabrication of metalenses. (a) Process flow of the 8‐inch S&R mold fabrication. (b) Photographs of 50 mm × 50 mm master (right) and 8‐inch S&R mold (left). (c) Process flow of the metalens fabrication with 8‐inch wafer‐level NIL. (d) Photographs of an 8‐inch wafer with fabricated metalenses.

By using the proposed S&R mold, an 8‐inch wafer‐level NIL process was successfully implemented as an alternative to DUV lithography and further integrated into the standard semiconductor manufacturing mass‐production line, incorporating core procedures, such as wafer cleaning, PECVD deposition of a‐Si and SiO_2_ layers. as well as ICP etching of SiO_2_ and a‐Si structures, as shown in Figure 2c (see the Experimental Section for details). The optical image of the 8‐inch wafer with fabricated metalenses can be seen in Figure 2d.

### 3.2 Optical Performances

To systematically evaluate the optical performance uniformity of the fabricated metalenses across the entire 8‐inch wafer, samples from different imprinting steps were selected for experimental characterization using the customized optical measurement setup illustrated in Figure 3a. This setup enables high‐precision detection of focal field distribution and transmission efficiency, ensuring reliable quantification of metalens performance. The measured results demonstrate that all tested metalenses exhibit excellent focusing capability, which is clearly validated by the normalized cross‐sectional intensity curves presented in Figure 3c. Notably, the intensity profiles of metalenses from different imprinting steps are nearly indistinguishable from each other and highly consistent with the designed curve. This consistency indicates that the proposed S&R imprinting process effectively minimizes process‐induced performance variations, a critical prerequisite for wafer‐level mass production.

**FIGURE 3:**
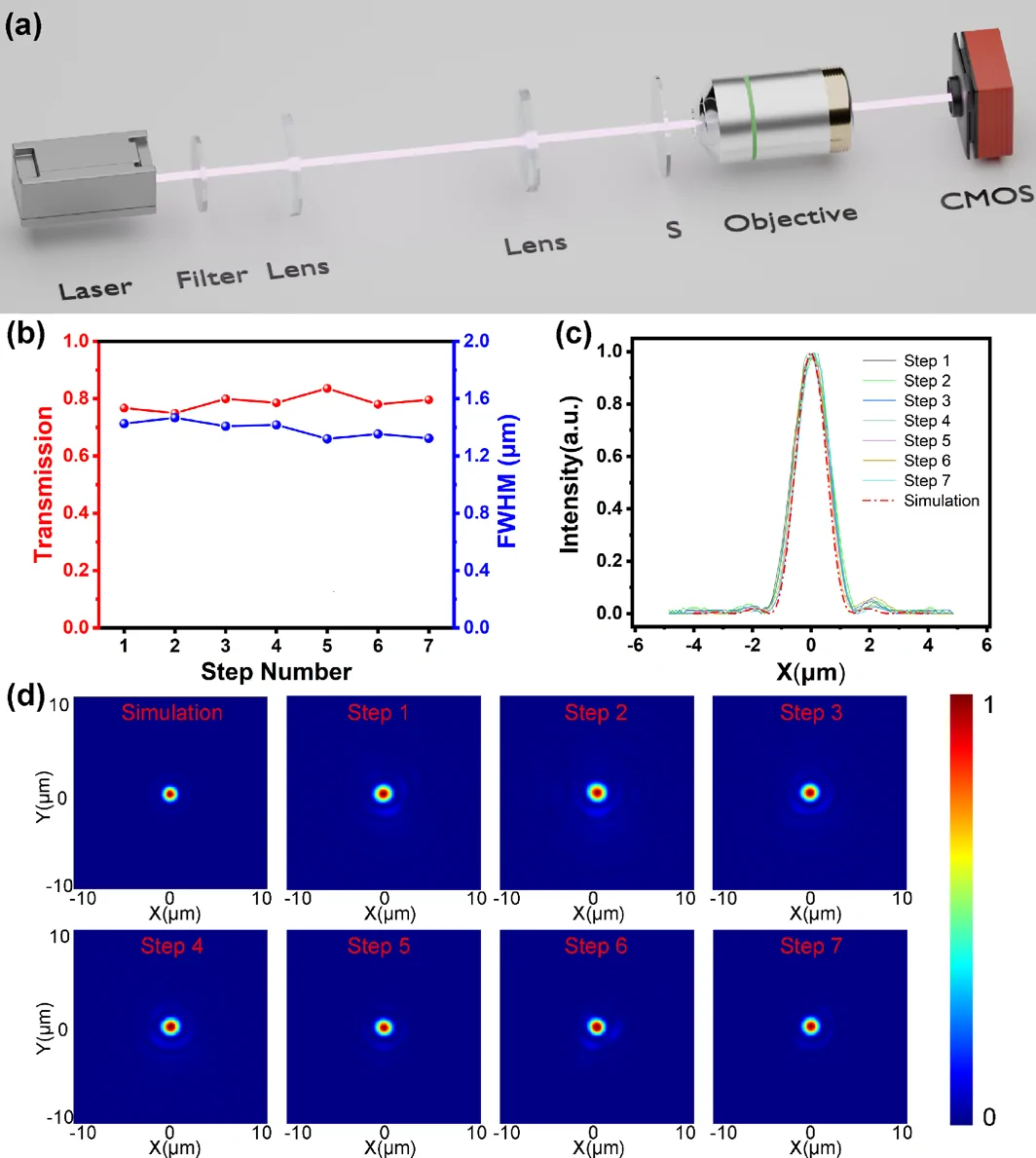
Optical characterization of the fabricated metalenses. (a) Schematic of the optical measurement setup. (b) Measured transmission (red curve) and FWHM (blue curve) of the metalens across Steps 1–7. (c) Normalized cross‐sectional intensity profiles of the metalens focal spot: comparison between simulated (red dashed curves) and measured results. (d) Normalized intensity distributions of the metalens in the *x–y* plane: comparison between simulated and measured results.

Quantitatively, the designed FWHM of the focal spot is 1.19 μm, which corresponds to the diffraction limit for the target near‐infrared wavelength and meta‐atom design parameters. The experimental measurement yields an average FWHM of 1.39 ± 0.05 μm (*n* = 7, representing seven independent metalenses sampled from each of the seven distinct imprinting steps), a value that is quite close to the diffraction limit. The marginal deviation from the designed FWHM can be attributed to slight alignment errors in the optical measurement system, whereas the intrinsic robustness of the FWHM against fabrication‐induced dimensional variations is theoretically validated by the tolerance analysis (Supporting Information S1: Figure S4). The simulated and measured normalized intensity distributions of the focal spots are compared in Figure 3d, revealing that the measured focal spot features high axial symmetry with no obvious distortion or side lobes. This good agreement between simulation and experiment confirms the rationality of the metalens design and the reliability of the fabrication process because axial symmetry is essential for applications requiring precise light focusing, such as LiDAR and AR/VR systems.

In addition to focusing performance, transmission efficiency is another key metric for practical metalens applications because it directly affects the signal‐to‐noise ratio and energy utilization of optical systems. The measured average transmission of all tested metalenses reaches 78.81 ± 2.73% (*n* = 7), a competitive value for high‐refractive‐index a‐Si based metasurfaces fabricated via NIL. As shown in Figure 3b, the transmission efficiencies of metalenses fabricated in different imprinting steps remain highly consistent, demonstrating excellent optical uniformity across the entire 8‐inch wafer. The negligible variation among the seven imprinting steps indicates that the optical performance is well preserved throughout the step‐and‐repeat fabrication process.

It is worth noting that the measured transmittance of the fabricated metalens is lower than the average transmittance predicted from simulations of isolated meta‐atoms. To evaluate the influence of fabrication‐induced dimensional deviations, additional simulations were performed by introducing a uniform 30 nm increase in the meta‐atom size (Supporting Information S1: Figure S5). The results show that the average transmittance decreases from approximately 95.0% to 91.2%. Further analysis reveals that the dimensional enlargement shifts a portion of the meta‐atoms away from their designed operating condition and induces resonance‐related transmission loss, particularly for meta‐atoms with radii near 130 nm. These results indicate that fabrication‐induced dimensional enlargement is an important contributor to the transmittance degradation.

However, dimensional deviations alone cannot fully account for the difference between the simulated and measured transmittance. In practical metalenses, additional loss mechanisms, including near‐field coupling between adjacent meta‐atoms, effective oblique‐incidence effects associated with large phase gradients, substrate loss, surface roughness, and parasitic absorption of the SiO_2_ hard mask, can further reduce the overall transmittance. Therefore, the lower transmittance of the fabricated metalens is attributed to the combined effects of fabrication‐induced dimensional deviations and device‐level optical losses. Future work will focus on optimizing the ICP etching process and the anti‐adhesion treatment of the S&R mold to further improve the optical performance of the metalens.

### 3.3 Structural Characterization

To analyze the dimension deviation of the meta‐atoms and its sources, the accuracy error of each key fabrication process is characterized. To evaluate dimensional variation of the S&R imprinting process, imprinted wafers were fabricated with both the master mold and S&R mold using identical NIL material and process parameters. Microscopy images as shown in Supporting Information S1: Figure S6 confirm the formation of complete, defect‐free metalenses on the imprinted wafer from the S&R mold. Figure 4a–c present SEM images of the imprinted metalens from the master mold at different magnifications, with four red markers indicating the measurement positions for each metalens under investigation. At each position, the diameters of the largest and smallest nano‐pillars were measured and quantified. Highly magnified SEM images of the imprinted wafer from the S&R‐mold are shown in Figure 4d–e. Detailed dimensional characterization across different imprinting steps is provided in Supporting Information S1: Figure S7, with the average nano‐pillar diameters summarized in Figure 4f.

**FIGURE 4:**
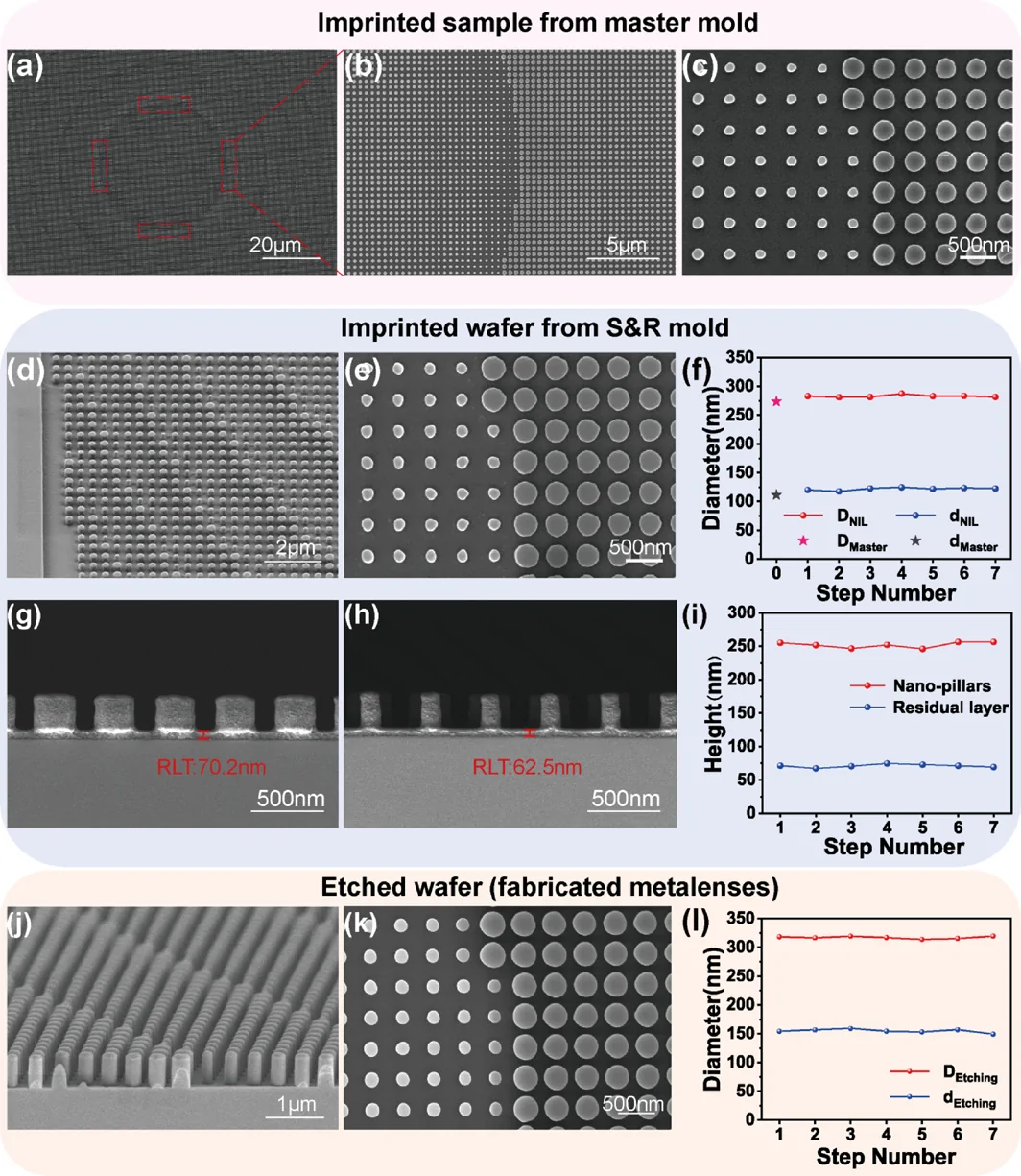
Characterizations of the metalenses. (a–c) SEM Images at different magnifications of the imprinted metalens via the master mold. (d, e) SEM Image of imprinted nano‐pillars with the S&R mold. (f) Chart of the average nano‐pillar diameters in different imprinting steps in the metalenses compared with the size in the master. (g, h) RLT for different nano‐pillar duty cycle. (i) The measured RLT and nano‐pillar heights across different imprinting steps. (j, k) Cross‐sectional SEM view of the etched meta‐atoms. (l) Chart of average meta‐atom diameters in different imprinting steps for metalenses on the wafer after etching.

For the master‐mold‐derived metalens, the mean diameter of the largest nano‐pillars (*D*_Master_) was 273.75 nm, and that of the smallest nano‐pillars (*d*_Master_) was 112.17 nm. For the S&R‐mold‐derived metalens, the mean diameters across all steps were *D*_NIL_ = 283.06 ± 1.97 nm (*n* = 28) and *d*_NIL_ = 121.99 ± 2.17 nm (*n* = 28). These small standard deviations demonstrate excellent dimensional consistency across the entire 8‐inch S&R mold and its imprinted wafer. Compared to the master mold, both large and small nano‐pillars fabricated via the S&R mold exhibit a ∼10 nm increase in diameter. This variation may arise from resist deformation and the several‐nanometer‐thick anti‐adhesion layer on the S&R mold. Given the consistent nature of this variation across all nano‐pillars, it can be readily compensated by modifying the design layout.

A thin, uniform residual layer in NIL samples is critical to ensuring high structural fidelity post‐etching. To optimize the RLT, a relatively high imprint pressure of 50,000 Pa was employed, while the resist spin‐coating speed was required to be varied and optimized. As shown in Supporting Information S1: Figure S8, different RLTs of 150, 70, and 45 nm were achieved at spin speeds of 2000, 3000, and 4000 rpm, respectively. Considering both demolding stability and RLT uniformity, a spin‐coating speed of 3000 rpm was selected as the optimized parameter for subsequent experiments.

RLT uniformity for different duty cycles of the meta‐atoms and for the entire 8‐inch wafer was investigated. As shown in Figure 4g,h, the RLT in the regions containing large or small nano‐pillars was ∼70.2 and ∼62.5 nm, respectively. Such small deviation of RLT (∼3% of the nano‐pillar's height) will not bring much influence on the etching performance, which can be demonstrated from the results after etching process, as presented in the next section of the paper. To evaluate the RLT uniformity across the entire 8‐inch wafer, SEM characterizations were conducted at seven distinct imprinting steps, as shown in Supporting Information S1: Figure S8. The RLT values and corresponding nano‐pillars’ heights across these steps are summarized in Figure 4i. The results show that across the entire wafer, the maximum variation in RLT for different imprinting steps is only ∼8 nm (∼3% of the nano‐pillars’ height). These results demonstrate that an excellent RLT uniformity can be achieved using the proposed S&R mold and NIL process for both different duty cycles and imprinting steps. It provides a robust foundation for the subsequent etching process.

The etching process is critical for precise transfer of imprinted patterns to underlying materials. The key challenge in wafer‐level fabrication is maintaining accuracy and uniformity of structural dimensions. As measured from the cross‐sectional SEM image shown in Figure 4j, the etched meta‐atoms exhibit near‐vertical sidewalls and a height of approximately 478 nm, which is close to the designed value of 475 nm. For direct comparison, the dimensions of identical meta‐atoms were systematically analyzed before and after etching. Figure 4k shows an SEM image of meta‐atoms at one imprinting step, with images of all seven steps provided in Supporting Information S1: Figure S9. All etched meta‐atoms remained intact, without observed collapse, missing features, or other defects. The mean diameters of the largest and smallest meta‐atoms after etching were 317.06 ± 1.97 nm (*n* = 28) and 154.95 ± 2.99 nm (*n* = 28), respectively. The small dimensional deviation across steps confirms high uniformity in the wafer‐level etching process.

Notably, meta‐atom diameters increased by ∼30 nm after etching, primarily due to the etching of the SiO_2_ hard mask. The etching system (LAM Research TCP 9400) used in this study is typically used for silicon etching with CD ≥ 300 nm, which is not ideally suitable for the present case. Two strategies may reduce this diameter deviation in future work: (1) employing an etching system suited for CDs of 120–300 nm; (2) directly reducing meta‐atom diameters in the design layout for compensation.

Figure 4f,i,l further quantify the dimensional uniformity of the meta‐atoms fabricated in different imprinting regions. The diameters of both the largest and smallest meta‐atoms, as well as the nanopillar height and residual‐layer thickness, remain highly consistent across all seven imprinting steps. After etching, the meta‐atom dimensions also show excellent agreement among different regions. No systematic dependence on the imprinting sequence is observed, and all dimensional variations remain within only a few nanometers over the entire 8‐inch wafer, demonstrating excellent field‐to‐field uniformity throughout the S&R fabrication process. The excellent agreement in both optical performance and structural dimensions indicates that each stitching region can be replicated and transferred with comparable fidelity during S&R mold fabrication and subsequent pattern transfer. The negligible field‐to‐field variations further demonstrate the robustness and reproducibility of the localized imprinting, UV exposure, and etching processes, validating the capability of the proposed S&R strategy to produce wafer‐scale metalens arrays with highly uniform optical and structural characteristics.

## 4 Conclusion

This study establishes a scalable, high‐yield route for wafer‐level metalenses, addressing the core cost bottleneck of NIL—large‐area master mold fabrication. Using a miniature EBL‐prepared master mold, an 8‐inch S&R mold was developed, realizing a sevenfold increase in metalens quantity with excellent structural fidelity and wafer‐scale performance uniformity. Integrating this S&R mold with standard semiconductor technologies, defect‐free NIL patterning was achieved on 8‐inch wafers, enabling the fabrication of high‐performance NIR all‐dielectric metalenses on quartz substrates. Characterizations confirm their structural and optical uniformity, with properties consistent with design specifications. Cost can be further reduced via master mold miniaturization and increased S&R cycles, while the a‐Si/quartz structure ensures high stability for harsh scenarios. This method exhibits broad material compatibility and excellent process scalability, making it readily extendable to the fabrication of visible‐region metalenses by using TiO_2_, Si_3_N_4_, or other transparent high‐index materials as meta‐atom materials. It provides a promising route for the low‐cost and scalable manufacturing of wafer‐scale metalenses. In future work, the proposed fabrication platform will be combined with advanced optical designs including aberration correction, achromatic design, and off‐axis optimization, to further develop application‐oriented metalenses for scenarios such as LiDAR and AR/VR.

## 5 Experimental Section

Fabrication of master mold: A master mold was fabricated on a 50 mm × 50 mm silicon substrate via EBL and ICP etching, with the fabrication processes illustrated in Supporting Information S1: Figure S2. Firstly, photoresist was spin‐coated onto the silicon substrate, followed by pre‐baking, patterning via the EBL tool (JEOL JBX‐9500FS system), development, and post‐baking to form the target photoresist pattern. Subsequently, the silicon substrate was etched to a depth of ∼250 nm using an ICP etching tool (OXFORD PlasmaPro 100 Cobra 180) with a SF_6_/CHF_3_ mixed gas. Finally, the residual photoresist was removed to obtain the master mold.

Fabrication of step‐and‐repeat mold: The S&R mold fabrication process is shown in Figure 2a.

1. Working stamp fabrication: First, an anti‐adhesive coating (AS01, OuGuangXin) was spin‐coated onto the previously prepared master mold. Afterward, the coated master mold was baked at 60°C for over 6 hours, followed by spin‐coating of a UV‐curable working stamp resist (WSA01, OuGuangXin). Subsequently, the master mold was transferred to a nanoimprinting system (GL4 R&D, GermanLitho) for automated imprinting, which proceeded as follows: (1) A pressure of 50,000 Pa was applied to laminate a polyethylene terephthalate (PET) flexible film onto the working stamp resist; (2) the working stamp resist was cured via exposure to a 365 nm LED UV lamp (intensity: 300 mW/cm^2^) for 5 minutes; and (3) the PET film with the cured working stamp pattern was peeled off from the master mold. Through these procedures, a working stamp with an inverse structure of the master mold was successfully obtained.
2. S&R mold fabrication: First, a layer of S&R imprinting resist (GL‐HS, GermanLitho) was spin‐coated onto an 8‐inch silicon wafer at 2000 rpm for 30s. The cured resist exhibits a Young's modulus of 650 MPa, providing sufficient mechanical stability for the subsequent processes. Subsequently, the wafer was transferred to an S&R imprinting system (GL300 SR‐F, GermanLitho) for the following processes: After presetting the imprinting position in the system software, the working stamp was moved to the initial position and pressed into the resist at 50,000 Pa. The resist was then cured via exposure to a 365 nm LED ultraviolet lamp (intensity: 300 mW/cm^2^) for 3 minutes. After curing, the working stamp was separated from the wafer and moved via a precision displacement stage to the next target position, repeating the aforementioned imprinting cycle. The S&R mold was fabricated through this step‐and‐repeat imprinting approach.
3. Anti‐adhesion treatment: After fabrication of the S&R mold, an approximately 3 nm‐thick Al_2_O_3_ layer was deposited on the mold surface by atomic layer deposition (ALD) to enhance its mechanical robustness and wear resistance. Subsequently, a fluorinated anti‐adhesion monolayer was deposited onto the Al_2_O_3_‐coated surface to reduce interfacial adhesion during the nanoimprint process.

Wafer‐scale NIL and etching: As shown in Figure 2c, the fabrication process for the 8‐inch working stamp is similar to that for the master mold described earlier, but using a mass‐production‐grade nanoimprinting system (GL200 CLIV, GermanLitho), including spin‐coating of the working stamp resist (WSA01) at 1000 rpm, imprinting under a pressure of 50,000 Pa, exposure to 365 nm UV light (intensity: 300 mW/cm^2^), and demolding. With the 8‐inch working stamp, the process flow proceeded as follows: first, a 475 nm‐thick a‐Si layer and a 150 nm‐thick SiO_2_ layer were deposited on a quartz wafer using a PECVD system, with TMS/SiH_4_/Ar and SiH_4_/N_2_O gas mixtures, respectively. Following deposition, the wafer was coated with an imprint resist (E301‐15, OuGuangXin) and pre‐baked on a hotplate at 80°C for 30 s. Subsequently, both the prepared wafer and the fabricated working stamp were loaded into a nanoimprinting system (GL200 CLIV, GermanLitho) for automated NIL processing, which included imprinting under 50,000 Pa pressure, UV curing for 3 minutes, and demolding. Finally, a mass‐production‐grade ICP etching system (TCP 9400, LAM Research) was used to etch the residual layer thickness (RLT) and the SiO_2_ hard mask via a CF_4_/CHF_3_ gas mixture, as well as the a‐Si layer via an O_2_/Cl_2_/C_2_F_6_ gas mixture.

The SEM images of the imprints were taken with a ZEISS GeminiSEM 360 and their feature dimensions were measured using ImageJ software.

Optical characterization: The optical performance of the metalenses was characterized using the optical system shown in Figure 3a. A laser beam (wavelength: 940 nm) was first collimated and expanded, then normally incident on the metalens. The intensity distribution of the focused light passing through the metalens was collected by an objective lens and imaged onto a CMOS camera (Thorlabs DCC1545M, pixel size = 5.2 μm), and the FWHM of the focal spot was calculated based on the acquired intensity distribution data. The transmittance of the metalenses was also measured using the same optical system.

## Author Contributions

**Wanjiao Zhang:** conceptualization, investigation, funding acquisition, writing – original draft, writing – review and editing, visualization, validation, methodology, software, formal analysis, project administration, resources, supervision, data curation. **Meng Wang:** data curation, supervision, resources, project administration, formal analysis, software, methodology, validation, visualization, writing – review and editing, writing – original draft, funding acquisition, investigation, conceptualization. **Yunxiao Cai:** conceptualization, investigation, funding acquisition, writing – original draft, writing – review and editing, visualization, validation, methodology, software, formal analysis, project administration, resources, supervision, data curation. **Qiu Wang:** conceptualization, investigation, funding acquisition, writing – original draft, writing – review and editing, visualization, validation, methodology, software, formal analysis, project administration, resources, supervision, data curation. **Yuan Wang:** conceptualization, investigation, funding acquisition, writing – original draft, writing – review and editing, visualization, validation, methodology, software, formal analysis, project administration, resources, supervision, data curation. **Lu Han:** resources, data curation, supervision, formal analysis, software, project administration, visualization, methodology, validation, writing – review and editing, writing – original draft, funding acquisition, investigation, conceptualization.

## Funding

The National Natural Science Foundation of China (Grant 62205253).

## Conflicts of Interest

The authors declare no conflicts of interest.

## Supporting information


Supporting Information S1

**Video S1:** Metalens focusing at different imprinting steps.

## Data Availability

The data that support the findings of this study are available from the corresponding author upon reasonable request.
