# Supplementary material for: Wafer‐Scale Near‐Infrared Metalenses With High Performance and Low Cost
Source: Nanophotonics. 2026 Aug 18;15(16):e70258. doi: 10.1002/nap2.70258 (PMC13672142; doi:10.1002/nap2.70258)
Supplement: Supplementary file 1 [file NAP2-15-e70258-s002.docx]

Supporting Information for

Wafer-Scale Near-Infrared Metalenses with High Performance and Low Cost

*Wanjiao Zhang, Meng Wang, Yunxiao Cai, Qiu Wang*, Yuan Wang* and Lu Han**

Wanjiao Zhang, Meng Wang, Yunxiao Cai, Lu Han

Hangzhou Institute for Advanced Study, University of Chinese Academy of Sciences, Hangzhou 310024, China

E-mail: hanlu@ucas.ac.cn

Qiu Wang

School of Information Engineering and the Hubei Key Laboratory of Broadband Wireless Communication and Sensor Networks, Wuhan University of Technology, Wuhan 430070, China

E-mail: wangqiu@whut.edu.cn

Yuan Wang

State Key Laboratory of Optical Quantum Materials, Department of Mechanical Engineering, The University of Hong Kong, Hong Kong 999077, China.
E-mail: yw25@hku.hk

**S1. Simulated polarization‑independent response of the meta‑atoms**

**Figure S1.** Simulated transmission and phase comparison curves of the meta‑atoms under x‑polarized (solid line) and y‑polarized (dashed line) incidence.

Figure S1 compares the simulated phase and transmittance responses of the cylindrical amorphous silicon meta-atoms under x- and y-polarized illumination at 940 nm. Owing to the rotational symmetry of the cylindrical geometry, the phase and transmittance curves for the two orthogonal polarization states almost completely overlap over the entire radius range. The results confirm the polarization-independent nature of the meta-atoms and indicate that the designed metalens can provide nearly identical wavefront modulation for different incident polarization states.

**S2. Master mold fabrication process**

**Figure S2.** Schematic of the master mold fabrication process.

**S3. Durability and anti‑adhesion layer stability of the S&R mold**

**Figure S3.** Durability characterization of the S&R mold before and after 50 imprint cycles. (a–e) SEM images after the 10th, 20th, 30th, 40th, and 50th imprint cycles, respectively. (f) Critical dimension as a function of imprint cycle number. (g) Water contact angle after initial anti‑adhesion treatment. (h) Water contact angle after 50 imprint cycles. (i) Water contact angle after re‑treatment of the anti‑adhesion layer.

To evaluate the durability of the S&R mold during repeated nanoimprinting, 50 consecutive wafer-level NIL cycles were performed using the same mold. Representative SEM images after the 10th to 50th imprinting cycles are shown in Figure S3a-e. No obvious structural degradation, collapse, or damage was observed after repeated imprinting and demolding. Quantitative measurements further show that the diameters of representative large and small nanopillars changed by less than 7 nm after 50 cycles (Figure S3f), indicating negligible mold wear and excellent structural stability.

The surface wettability of the mold was characterized by water contact-angle measurements, as shown in Figure S3g and S3h. The contact angle decreased slightly from 107.4° after the first imprinting cycle to 103.8° after the 50th cycle, while remaining above the threshold required for reliable demolding under our imprinting conditions. This result indicates that the mold retained good anti-adhesion performance throughout repeated use. Notably, a water contact angle of 103.8° is still sufficient for reliable demolding; the subsequent re-deposition was performed to verify recoverability rather than because the mold had failed.

The durability of the S&R mold originates from the combined effects of an ALD-grown Al₂O₃ protective layer and a fluorinated anti-adhesion monolayer. The Al₂O₃ layer enhances the mechanical robustness and wear resistance of the nanostructured surface, whereas the fluorinated monolayer reduces interfacial adhesion during demolding. After 50 imprinting cycles, the anti-adhesion layer was re-deposited on the mold surface. As shown in Figure S3i, the water contact angle recovered to 107.5°, approaching its initial value. This suggests that the gradual decrease in demolding performance mainly results from degradation of the anti-adhesion layer rather than structural damage to the mold itself. Therefore, the service lifetime of the mold can be further extended through periodic renewal of the anti-adhesion layer.

**S4 Fabrication-tolerance analysis**

**Figure S4.** Focal length and full width at half maximum (FWHM) of the metalens under different critical dimension (CD) deviations.

To further evaluate the fabrication tolerance of the proposed metalens design, additional simulations were performed by introducing uniform critical-dimension (CD) deviations ranging from −40 nm to +40 nm. The resulting focal length and full width at half maximum (FWHM) of the focal spot are summarized in Figure S4.

As shown in Figure S4, the focal spot size remains nearly unchanged throughout the entire CD deviation range. The simulated FWHM is maintained at approximately 1.19 μm, indicating that the focusing quality is largely insensitive to uniform dimensional variations. The focal length also exhibits excellent stability. Within a CD deviation range of ±20 nm, the focal length remains essentially constant. Even when the dimensional deviation reaches ±40 nm, the focal length changes by less than 1.6 μm, corresponding to a relative variation of only ~0.35%.

The observed robustness originates from the fact that the experimentally observed dimensional deviation mainly manifests as a global enlargement or reduction of the meta-atom dimensions rather than random local variations. Although such uniform dimensional deviations modify the absolute phase response and transmission efficiency of individual meta-atoms, they introduce only limited perturbation to the overall phase distribution of the metalens[1-3]. Consequently, the focusing characteristics remain largely preserved.

These results indicate that the proposed metalens design possesses a relatively high tolerance to fabrication-induced dimensional deviations. This finding is consistent with the simulations presented in Figure S5, where a 30 nm dimensional enlargement leads to a measurable reduction in transmission efficiency but causes negligible change in the focal position. The tolerance analysis therefore provides a reasonable explanation for the good agreement observed between the experimentally measured and simulated focal spots despite the presence of fabrication errors.

**S5. Tolerance simulation analysis of the metalens under +30 nm critical dimension deviation**

**Figure S5.** Simulated optical performance of the metalens under a +30 nm critical dimension (CD) deviation. (a) Phase of the meta-atoms as a function of radius for the original design (red) and with +30 nm CD deviation (blue). (b) Transmittance of the meta-atoms as a function of radius for the original design (red) and with +30 nm CD deviation (blue). (c) Radial transmittance comparison along the metalens aperture: original design (red) and simulation with +30 nm CD deviation (blue). (d) Simulated electric field intensity distribution in the *x-z* plane for the metalens with +30 nm CD deviation.

To quantitatively evaluate the influence of fabrication-induced dimensional deviations on the optical performance of the meta-atoms, simulations were performed for both the original design and a modified structure with the diameter increased by 30 nm. As shown in Figure S5a, the phase response of the original meta-atoms varies smoothly and monotonically over the radius range of 60–140 nm, providing nearly full 2π phase coverage. After introducing a 30 nm diameter increase, a pronounced phase discontinuity appears near the original radius of approximately 130 nm, indicating the excitation of a strong resonant mode.

The corresponding transmittance responses are shown in Figure S5b. For meta-atoms with original radii between 60 and 120 nm, the transmittance remains above 90% even after dimensional enlargement. In contrast, for meta-atoms with radii between approximately 120 and 140 nm, the transmittance decreases significantly due to resonance-induced transmission loss associated with the enlarged dimensions.

To further evaluate the impact of fabrication errors on the entire metalens, the transmittance of all meta-atoms distributed from the center to the edge of the metalens was calculated, as shown in Figure S5c. Compared with the original design, the enlarged meta-atoms exhibit pronounced transmittance degradation at several specific locations (corresponding to the radius range of approximately 135-155 nm where the transmittance of enlarged meta-atoms drops significantly in Figure S5b), whereas most meta-atoms are only weakly affected. Statistical analysis shows that the average transmittance decreases from approximately 95.0% in the original design to 91.2% after introducing a 30 nm dimensional deviation, corresponding to a reduction of about 3.8%. These results indicate that fabrication-induced enlargement of the meta-atom dimensions is a quantitatively identifiable contributor to the transmittance degradation of the fabricated metalens.

The experimentally measured average transmittance of the fabricated metalens is 78.8%, which remains substantially lower than the simulated value of 91.2% even after accounting for the 30 nm dimensional deviation. This observation indicates that fabrication-induced dimensional errors alone cannot fully explain the transmittance degradation. Additional loss mechanisms are therefore expected to contribute to the discrepancy. These include the intrinsic transmission loss of the quartz substrate (approximately 2.2% as measured experimentally), near-field coupling between neighboring non-periodic meta-atoms, effective oblique-incidence effects associated with the large phase gradients near the edge region of the metalens, and fabrication-related losses such as slight over-etching or under-etching of the a-Si nanopillars, surface and sidewall roughness, and parasitic absorption introduced by the SiO₂ hard mask[3-7]. The combined effects of these factors further reduce the overall transmittance of the fabricated metalens.

The influence of dimensional deviations on the focusing performance was also evaluated. As shown in Figure S5d, the simulated focal position remains nearly unchanged after introducing the 30 nm dimensional deviation, indicating that the focusing functionality of the metalens is largely preserved. This simulation result is consistent with both the experimental measurements and the focusing demonstrations obtained from different wafer locations (Supplementary Video S1). These combined results confirm that the proposed metalens design exhibits good tolerance to fabrication-induced dimensional deviations, maintaining stable focusing performance despite a measurable reduction in transmission efficiency.

**S6. Confocal microscopy images of imprinted wafer from the 8-inch S&R mold at different imprinting steps**

**Figure S6.** Macroscopic characterisation of metalenses: (a–g) Metalenses fabricated at different positions on the imprinted wafer from the 8-inch S&R mold (steps 1–7)

Figure S6 presents the macroscopic characterization results obtained via a confocal microscope, including the metalenses at the corresponding positions across 7 imprinting steps of the S&R mold. The results show that for the large-sized mold fabricated by step-and-repeat nanoimprint lithography, the macrostructures of the metalenses exhibit no large-area defects and excellent structural integrity. This confirms the feasibility of the large-sized metalens mold fabrication method proposed in this study.

**S7. SEM images of imprinted wafer from the 8-inch S&R mold at different imprinting steps**

**Figure S7.** Microscopic characterisation of the cylinder nanostructures on the 8-inch S&R mold. (a–f) SEM images of the cylinder nanostructures fabricated at different positions on the S&R mold (steps 2–7).

Figure S7 presents the microscopic characterization results of the S&R mold via SEM, which was used to characterize the critical dimensions of the cylinder nanostructures across different imprinting steps (where the SEM micrograph of Step 1 corresponds to Figure 4e in the main text). The selection of test areas and the cylinder nanostructures is consistent with that in the main text. The results align with the *D*_NIL_ and *d*_NIL_ curve data in Figure 4f, and the critical dimensions of the cylinder nanostructures across different steps exhibit excellent consistency. These results fully confirm that the fabrication method proposed in this study has outstanding process consistency and structural fidelity, verifying the reliability of the method itself.

**S8. SEM images of residual layer thickness for 8-Inch S&R mold imprints at different steps**

**Figure S8.** SEM characterizations of RLT from NIL. (a) RLT with varied spin-coating speed. (b-h) RLT of the 8-inch S&R mold imprint corresponding to steps 1-7, respectively.

Figures S8b-h sequentially present SEM characterization results of RLT on the fabricated 8-inch S&R mold after nanoimprinting. During SEM measurement, metalenses at identical positions across seven steps were tested to comprehensively evaluate the uniformity of RLT. The measured results consistently demonstrate excellent thickness uniformity of RLT, further validating the high uniformity of the nanoimprinting process and the flatness of the S&R mold surface.

**S9. SEM images of the metalenses on the 8-inch wafer after etching at different steps**

**Figure S9.** Microscopic characterisation of the metalenses: (a–f) SEM images of the fabricated metalenses after etching at different positions for size characterization, corresponding to steps 2–7, respectively.

Figure S9 presents the SEM characterization results of the critical dimensions (CD) of metalenses after etching, where the SEM micrograph of Step 1 corresponds to Figure 4k in the main text. The results show that the CD values of metalenses across different steps on the etched wafer exhibit high consistency, confirming that the etching process employed in this study has excellent stability. It should be noted that there is a certain deviation in the final CD values compared with those of the original master mold, and this phenomenon is primarily attributed to the influence of etching process equipment. However, such deviations can be effectively eliminated through subsequent process optimization.

**References**

[1] Zhou Y, Hu T, Li Y, et al. A performance study of dielectric metalens with process-induced defects[J]. IEEE Photonics Journal, 2020, 12(3): 1-14.
[2] Dirdal C A, Jensen G U, Angelskår H, et al. Towards high-throughput large-area metalens fabrication using UV-nanoimprint lithography and Bosch deep reactive ion etching[J]. Optics Express, 2020, 28(10): 15542-15561.
[3] Liu X, Chen R, Lu Y, et al. Optical performances of near-infrared metalenses with process-induced defects[J]. Chinese Optics Letters, 2025, 23(6): 063602.
[4] Wu Z, Huang X, Yu N, et al. Inverse design of a dielectric metasurface by the spatial coupled mode theory[J]. ACS Photonics, 2024, 11(8): 3019-3025.
[5] Bae M, Jang E, Kang C, et al. Inverse Design of Thermal Imaging Metalens Achieving 100° Field of View on a 4× 4 Microbolometer Array[J]. Micromachines, 2025, 17(1): 65.
[6] Arbabi A, Arbabi E, Mansouree M, et al. Increasing efficiency of high numerical aperture metasurfaces using the grating averaging technique[J]. Scientific Reports, 2020, 10(1): 7124.
[7] Chung H, Miller O D. High-NA achromatic metalenses by inverse design[J]. Optics Express, 2020, 28(5): 6945-6965.
